# Combining ethanol and cocaine exposure during adolescence increased cocaine reward sensitivity in adult rats as evaluated by a single exposure place preference paradigm

**DOI:** 10.3389/fphar.2025.1668957

**Published:** 2025-09-01

**Authors:** Carles Colom-Rocha, M. Julia García-Fuster

**Affiliations:** ^1^ IUNICS, University of the Balearic Islands, Palma, Spain; ^2^ Health Research Institute of the Balearic Islands (IdISBa), Palma, Spain; ^3^ Department of Medicine, University of the Balearic Islands, Palma, Spain; ^4^ Red de Investigación en Atención Primaria en Adicciones (RIAPAd), ISCIII, Madrid, Spain

**Keywords:** adolescence, polysubstance use, vulnerability to cocaine addiction, biological sex, validating new rodent models

## Abstract

There is an urgent need for better understanding the long-term impact of polysubstance use initiation during adolescence. Our preclinical study examined the combined impact of ethanol and cocaine during a vulnerable window in adolescence on the long-term changes on cocaine reward emerging throughout withdrawal in adulthood. Male and female Sprague-Dawley rats were treated in adolescence with non-contingent paradigms of ethanol, cocaine, their combination or vehicle. Cocaine reward was assessed in adult rats, following prolonged withdrawal, by a simple paradigm based on a single exposure place preference previously characterized in mice, which was validated for Sprague-Dawley male and female adult rats. The main results demonstrated an increased impact of combining drugs (ethanol with cocaine) during adolescence on the outcome observed in adulthood, since a single exposure to cocaine increased the rewarding response induced by cocaine in these animals (i.e., increased time spent in the cocaine paired compartment). The combination of drugs during adolescence seemed to overcome that initial lack of response observed by just one of the drugs, thus presenting an augmented risk-factor for later consequences in adult rats of both sexes. To conclude, an early adolescent combined drug experience proved clear consequences on the emerging addictive-like behaviors observed in adulthood for both sexes.

## Introduction

There is an urgent need for better understanding the long-term consequences of polysubstance use initiation during adolescence and how this combined exposure might impact the expected addictive-like vulnerability induced by early drug exposure. Although great progress has been made at the preclinical level evaluating the impact of the combined used of alcohol, cannabis and nicotine (Crummy et al., 2020; Steinfeld et al., 2023), few studies modeled the combined early exposure of alcohol and cocaine in adolescence (e.g., Colom-Rocha et al., 2024). In fact, previous reports mainly assessed the cardiotoxic effects mediated by cocaethylene (the active metabolite produced by both drugs), leaving room for novel studies on the long-term addictive-like vulnerability caused by the combined early exposure of alcohol and cocaine during adolescence. Remarkably, the use of rodents offers a good preclinical model (García-Fuster, 2021; Nieto et al., 2021), with face and construct validity (Spear, 2004), in which to evaluate how adolescent vulnerability (i.e., polydrug exposure) impacts the development of adult psychopathology (e.g., Adriani and Laviola, 2004).

Alcohol is one of the most used recreational drugs in the world, with a large group of consumers initiating its use in adolescence (reviewed by Steinfeld et al., 2023). This is backed up by the yearly reports from the European Monitoring Centre for Drugs and Drug Addiction (EMCDDA), and the US National Center for Drug Abuse Statistics (NCDAS), stating that alcohol is by far the most consumed substance among teens and young adults, with a lifetime alcohol consumption of 73% of adolescents (i.e., data from the 2024 European School Survey Project on Alcohol and Other Drugs, ESPAD). Moreover, 13% of students reported having used an illicit drug at least once in their lifetime, with an average prevalence of cocaine use among adolescents of 2.3%, the second most used illicit drug after cannabis. Out of this prevalence, 5% of these young individuals who reported heavy alcohol use in the past month also consumed cocaine as reported in ESPAD (Graziani et al., 2014), and since alcohol initiation generally starts sooner (i.e., at age 13 or younger), the combined use of these drugs generally parallels the time at which cocaine’s use starts, since at age 13 or younger, only an average of 0.9% of adolescents consume cocaine. These data suggested that although the first experiences with alcohol start in early adolescence, its potential combination with cocaine might not initiate until mid-adolescence. Accordingly, and given the lack of preclinical reports studying the long-term impact of early polydrug exposure in adolescence on addictive-like vulnerability behaviors in adulthood, we aimed at combining alcohol and cocaine at a vulnerable window during adolescence to study its outcome.

In this scenario, our way of modeling in rodents the combined early exposure of alcohol and cocaine centered in a window of adolescent vulnerability previously characterized by our group (e.g., García-Cabrerizo et al., 2015; García-Cabrerizo and García-Fuster, 2016; García-Fuster et al., 2017; García-Cabrerizo and García-Fuster, 2019), including the polydrug exposure of alcohol and cocaine and its later addictive-like liability in adulthood (i.e., voluntary ethanol consumption; see Colom-Rocha et al., 2024). In particular, the window selected for treatment covered a period between early (postnatal day, PND, 21-34) to mid (PND 34-46) adolescence (Spear, 2004), starting on PND 29 and up to PND 38 (Colom-Rocha et al., 2024), thus paralleling the stages observed in humans (i.e., early: from 10 to 13 years, middle: from 14 to 17 years; Christie and Viner, 2005; Backes and Bonnie, 2019), and aiming at mimicking the earlier exposure to alcohol than cocaine. Drugs administered at this window of vulnerability impacted affective- (García-Cabrerizo and García-Fuster, 2019) and addictive-like behaviors (García-Fuster et al., 2017; Parsegian et al., 2022; Colom-Rocha et al., 2024) after drug re-exposure in adulthood, as well as induced signs of neurotoxicity (García-Cabrerizo et al., 2015; García-Cabrerizo and García-Fuster, 2016; García-Fuster et al., 2017; Parsegian et al., 2022), laying out clear consequences induced by early drug initiation.

In the present follow-up study, we therefore aimed at evaluating how the combination of alcohol and cocaine exposure during this specific window of adolescence (see Colom-Rocha et al., 2024) might impact cocaine reward as assessed in adult rats of both sexes by a single exposure place preference paradigm. To evaluate changes in the initial rewarding effects of cocaine, in our first objective, we adapted a recent protocol that proved initial rewarding effects of cocaine (Runegaard et al., 2017) or amphetamine (Runegaard et al., 2019) based on a single exposure place preference procedure in mice (Runegaard et al., 2017), to adult Sprague-Dawley rats of both sexes. This method offered a rapid, reliable and useful approach to evaluate cocaine reward in a novel environment, without habituation or initial preference testing, and that avoided the need for repeated drug injections (Runegaard et al., 2017). In this context, the second and main objective of our study was to evaluate the long-term impact of adolescent exposure to alcohol, cocaine or their combination administered during a vulnerable developmental window on the rewarding properties of cocaine in adulthood following persistent withdrawal.

## Methods

### Animals

**FIGURE 1:**
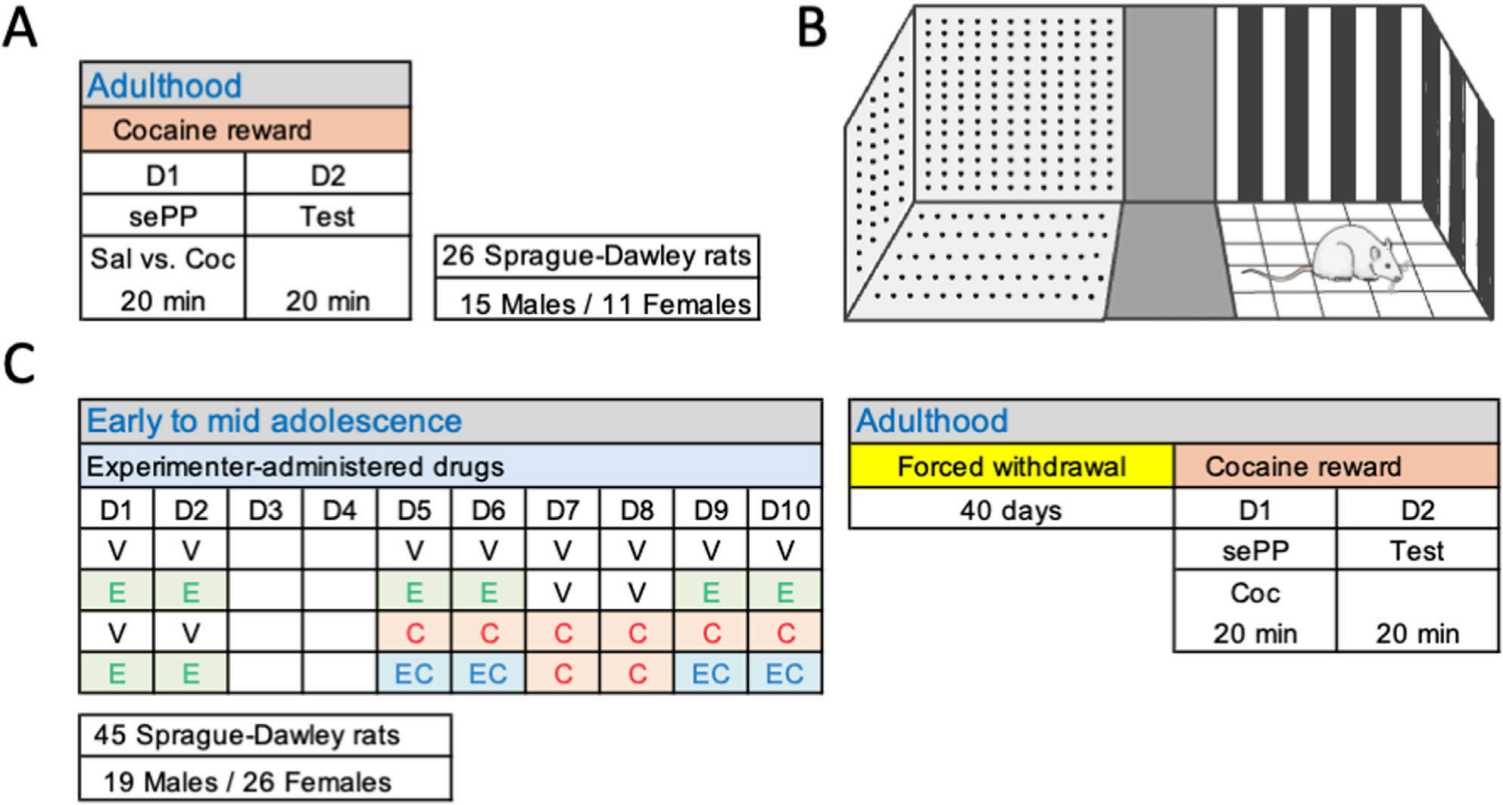
Experimental timeline. **(A)** Cocaine reward in 26 adult Sprague-Dawley rats as assessed by a single exposure place preference (sePP) paradigm. The protocol was conducted over 2 days: exposure session (D1) and test (D2), for 20 min each day (see more details in the methods section). **(B)** Schematic representation of the conditioning apparatus (67.5 × 24 cm, 40 cm high, without lid) which was distributed in three sections: a neutral zone (11.5 × 24 cm) in the middle, separating two compartments (28 × 24 cm) with different wall patterns and floor textures. Two dividers (24 × 40 cm) could be inserted between the neutral zone and the two outer compartments to isolate the paired compartment. **(C)** Impact of adolescent drug exposure on cocaine reward as assessed by a sePP paradigm in 45 Sprague-Dawley rats. Following the adolescent treatment (V, vehicle; E, ethanol; C, cocaine; EC, combined ethanol and cocaine) and 40 days of forced withdrawal, cocaine reward was assessed in all rats by the same 2-day protocol: exposure session (D1) and test (D2), for 20 min each day (see more details in the methods section).

For this investigation we used a total of 71 Sprague-Dawley rats of both sexes in two independent experiments (26 and 45 respectively; Figure 1). Rats were bred in the animal facility at the University of the Balearic Islands; after weaning (post-natal, PND, 21) rats were housed in standard cages (groups of 2-4 rats per sex) under controlled environmental conditions: 22 °C, 70% humidity, 12 h light/dark schedule, lights on at 08:00 h, with unlimited access to food and water. Experimental procedures were performed during the light period and were design following ARRIVE Guidelines (Percie du Sert et al., 2020), EU Directive 2010/63/EU, and Spanish Royal Decree 53/2013 (with prior approval from the Local Bioethical Committee: CEEA 148-09-20, and the Regional Government: 2021/01/AEXP). The specific stages of the estrous cycle were not monitored as previously suggested for experimental procedures in which the hormonal cyclicity is not part of the research question (e.g., Becker et al., 2016; Beltz et al., 2019; Kaluve et al., 2022), and to minimize the number of unnecessary procedures to which rats were exposed and their suffering.

### Cocaine reward in adult rats assessed by a single exposure place preference paradigm

To evaluate changes on the initial rewarding effects of cocaine we adapted a recent protocol based on the use of a single exposure to cocaine to induce place preference in mice (Runegaard et al., 2017; Runegaard et al., 2019). Given the simplicity of the method, our first aim was to reproduce the paradigm on 26 adult Sprague-Dawley rats of both sexes (Figure 1A). The conditioning apparatus (67.5 × 24 cm, 40 cm high, without lid) was distributed in three sections: a neutral zone (11.5 × 24 cm) in the middle that separated two compartments (28 × 24 cm) with different wall patterns and floor textures (Figure 1B), as previously described (Jornet-Plaza et al., 2025). The place preference protocol was conducted over 2 days, with drug exposure on D1 and testing on D2. On both days, rats were allowed to acclimatize for at least 1 h to the experimental room prior to the procedure. On D1, rats received a single i. p. injection of either saline (0.9% NaCl, n = 12) or cocaine (15 mg/kg, n = 14), which was administered at 1 mL/kg, and was paired in a counterbalance manner in alternate compartments by experimental group and sex (Figures 1A,B). The dose of cocaine was selected from prior experiments utilizing this protocol in mice (Runegaard et al., 2017; Runegaard et al., 2019), and/or protocols to induce psychomotor sensitization in rats (García-Fuster et al., 2010; García-Fuster et al., 2017). The compartments were isolated by two dividers (24 × 40 cm) inserted between the neutral zone and the two outer compartments, in which each rat was confined right after the specific treatment injection (either cocaine or saline) for a duration of 20 min. On D2, dividers were removed, and rats were allowed to freely explore all compartments during 20 min (Figures 1A,B). Sessions were videotaped and results were analyzed with a video-tracking software (SMART Video Tracking System, version 3.0.; Panlab, Harvard Apparatus; Barcelona, Spain) to calculate the time spent in each of the three compartments (paired, neutral, unpaired), the number of entries in each compartment, as well as the total distance travelled (cm). The % time in the drug-paired compartment was calculated in relation to the time spent in the paired vs. unpaired compartment.

### Impact of adolescent drug exposure on cocaine reward as assessed by a single exposure place preference paradigm in adulthood

For the next experiment, we utilized this single-cocaine exposure place preference paradigm to evaluate how early drug exposure in adolescence might change cocaine reward in adulthood (Figure 1C). To do so, 45 Sprague-Dawley rats were treated following a non-contingent regimen of drug exposure (i.p., 2 mL/kg) during adolescence (starting on PND 29, D1, and up to PND 38, D10, Figure 1C) with ethanol, cocaine or a combination of both drugs following a previous procedure (Colom-Rocha et al., 2024). Ethanol administration started earlier than cocaine during adolescence, on D1 (PND 29), in line with the consumption pattern observed in adolescents, and followed a binge intermittent design (2 g/kg prepared from a solution of 30% ethanol; 3 rounds of 2 days at 48-h intervals on D1-D2, D5-D6 and D9-D10; Figure 1B) as previously described Colom-Rocha et al., 2024, and references within). Note that ethanol was administered i. p. instead of oral gavage so it could be combined with cocaine as detailed below in the combination procedure (Figure 1C). Cocaine administration followed a psychomotor sensitizing regimen exposure extensively characterized in adolescence by our research group (García-Cabrerizo et al., 2015; García-Cabrerizo and García-Fuster, 2016; García-Fuster et al., 2017; García-Cabrerizo and García-Fuster, 2019; Parsegian et al., 2022; Colom-Rocha et al., 2024), based on a daily dose of 15 mg/kg of cocaine (prepared in vehicle solution) starting on PND 33 during mid adolescence and lasting 6 consecutive days (D5-D10; Figure 1C). On the days particular some rats needed to receive the combination of ethanol and cocaine (EC as shown in Figure 1C), the specific amount of cocaine was added to the ethanol solution, so rats received just one injection with the correct dose of both drugs. The vehicle solution (0.9% NaCl) was administered at the indicated times to control rats (D1-D2 and D5-D10), but also to rats from other experimental groups when programmed (D1-D2 for cocaine groups and D7-D8 for ethanol groups; Figure 1C) (Colom-Rocha et al., 2024). All procedures and pharmacological administrations were performed by the same experimenter.

Rats were then left undisturbed for up to 40 days (forced drug withdrawal) until adulthood when they were exposed to the single-exposure place preference procedure (Figure 1C). The place preference protocol was also conducted over 2 days, with the exposure session on D1 and testing on D2. Like the previous experiment, rats were daily allowed to acclimatize for at least 1 h to the experimental room. Since we have already established that cocaine induced reward following a single exposure in the first experiment, to not replicate groups and to reduce the number of animals used, all rats from this experiment only received a single dose of cocaine (15 mg/kg, i. p.) on D1. Cocaine administration was paired in a counterbalance manner in alternate compartments in which rats were confined right after drug injection for a duration of 20 min. On D2, dividers were removed, and rats were allowed to freely explore all compartments during 20 min (Figure 1C). Sessions were videotaped and results were analyzed with a video-tracking software (SMART Video Tracking System, version 3.0.; Panlab, Harvard Apparatus; Barcelona, Spain) to calculate the time spent in each of the three compartments (paired, neutral, unpaired), the number of entries in each compartment, as well as the total distance travelled (cm). The % time in the drug-paired compartment was calculated in relation to the time spent in the paired vs. unpaired compartment.

### Data analyses and statistics

**FIGURE 2:**
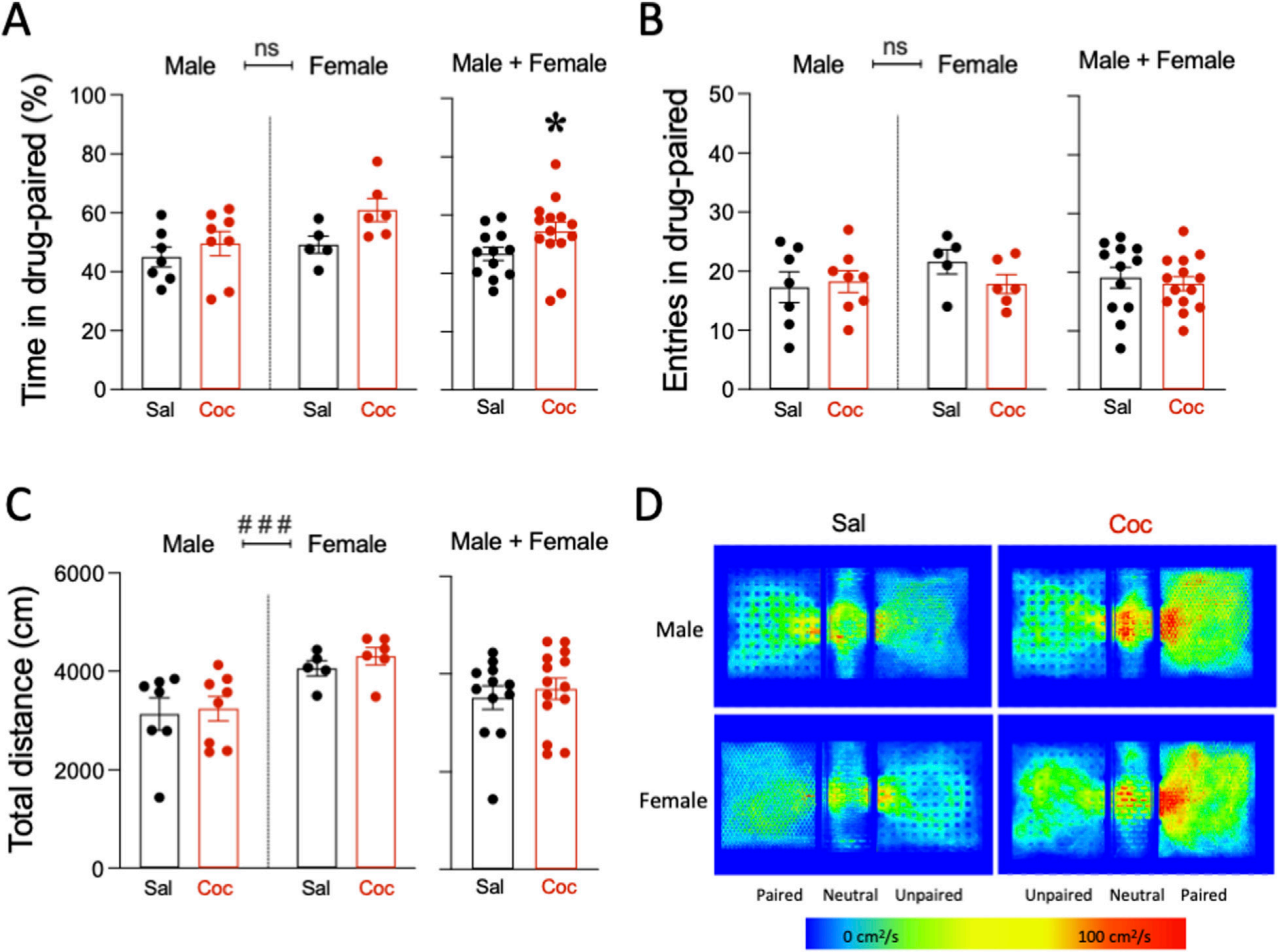
A single exposure place preference paradigm induced cocaine reward in adult rats. **(A)** Time spent in drug-paired (%), **(B)** entries in drug-paired (number), and **(C)** total distance (cm) in the single exposure place preference (sePP) paradigm in adult rats challenged with a single dose of cocaine (15 mg/kg) or saline (0.9% NaCl). Columns represent the mean ± SEM of the corresponding measurement, with individual values shown as symbols for each rat. Groups of treatment: vehicle-male (n = 9); ethanol-male (n = 9); cocaine-male (n = 9); ethanol + cocaine-male (n = 7); vehicle-female (n = 6); ethanol-female (n = 9); cocaine-female (n = 9); ethanol + cocaine-female (n = 8). Two-way ANOVAs (independent variables: treatment and sex) were performed (overall effect of sex: ^###^ *p* < 0.001 when comparing female vs. male rats). Student’s t-test were performed when combining male and female rats and analyzing the results independently of sex. *p < 0.05 vs. saline-challenged rats. **(D)** Representative heatmaps of the spatial exploration rate (i.e., area travelled per second during the test as expressed in cm^2^/s) for each compartment during the sePP assays in adult rats on D2. Both male and female rats showed increased cocaine (Coc) preference during the test session as compared with rats injected with saline (Sal).

**FIGURE 3:**
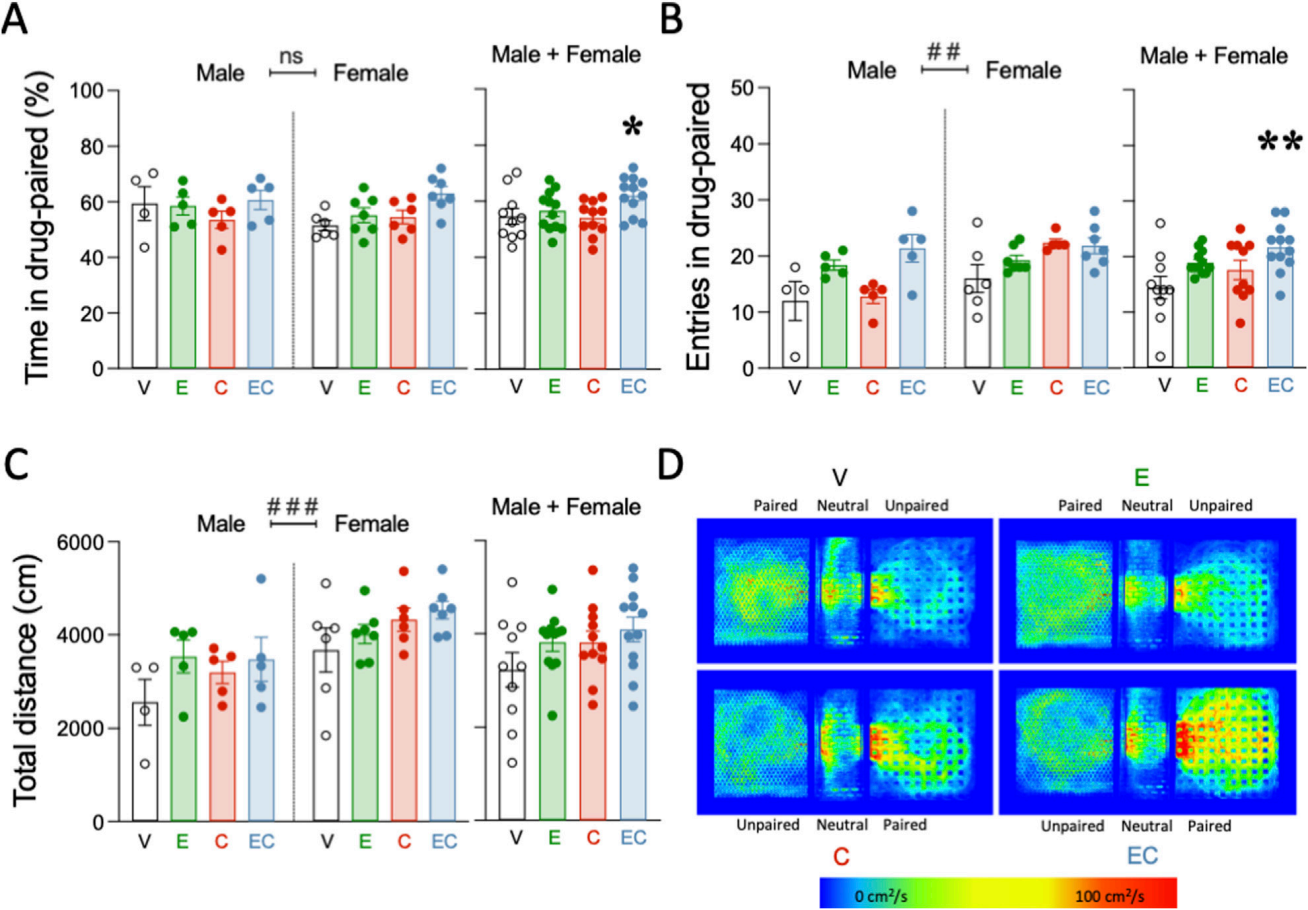
Adolescent polydrug exposure increased cocaine reward in adulthood as assessed by a single exposure place preference paradigm. **(A)** Time spent in cocaine-paired (%), **(B)** entries in cocaine-paired (number), and **(C)** total distance (cm) in the single exposure place preference (sePP) paradigm in adult rats challenged with a single dose of cocaine (15 mg/kg). Columns represent the mean ± SEM of the corresponding measurement, with individual values shown as symbols for each rat. Groups of treatment: vehicle (V)-male (n = 4); ethanol (E)-male (n = 5); cocaine (C)-male (n = 5); ethanol + cocaine (EC)-male (n = 5); V-female (n = 6); E-female (n = 7); C-female (n = 6); EC-female (n = 7). Two-way ANOVAs (independent variables: adolescent treatment and sex) were performed (overall effect of sex: ^###^ *p* < 0.001 when comparing female vs. male rats). One-way ANOVAs were performed when combining male and female rats and analyzing the results independently of sex. **p < 0.01 and *p < 0.05 vs. vehicle-treated rats during adolescence. **(D)** Representative heatmaps of the spatial exploration rate (i.e., area travelled per second during the test as expressed in cm^2^/s) for each compartment during the sePP assays in adult rats of each treatment group (V, E, C, EC) on D2. Rats treated with a combination of ethanol and cocaine (EC) during adolescence showed increased cocaine (Coc) preference during the test session as compared to rats treated with vehicle (V).

All graph plotting and data analyses were done with GraphPad Prism, Version 10 (GraphPad Software, United States) following guidelines in experimental pharmacology for displaying data and statistical methods (Michel et al., 2020). Results are reported as mean values ±standard error of the mean (SEM); individual symbols are shown for each rat within bar-graphs. Assumptions for normality of data distribution and homogeneity of variance were met. Data was analyzed through two-way ANOVAs (with sex and treatment as study variables), or independently of biological sex through unpaired *t*-tests (Figure 2) or one-way ANOVAs (Figure 3). Dunnett’s multiple comparisons tests were performed when appropriate. Statistical significance was set at *p* ≤ 0.05. Data supporting the present findings will be available upon reasonable request to the corresponding author.

## Results

### Cocaine reward in adult rats as observed by a single exposure place preference paradigm

A single cocaine exposure was capable of inducing place preference in adult rats as compared to rats injected with a saline solution (Figure 2). The results for the time spent in the drug-paired compartment (saline vs. cocaine-paired chamber) showed a significant effect of treatment (F_1, 22_ = 4.50, *p* = 0.045), and no effect of sex (F_1, 22_ = 4.08, *p* = 0.056) or treatment × sex interaction (F_1, 22_ = 0.88, *p* = 0.359; Figure 2A). When combining rats of both sexes, cocaine increased the time rats spent in the drug-paired compartment (+7.7 ± 4.1%, *t* = 1.90, *df* = 24, *p = 0.035 vs. saline-treated rats; Figure 2A). No changes were observed when analyzing the number of entries in the paired compartment (treatment: F_1, 22_ = 0.43, *p* = 0.520; sex: F_1, 22_ = 0.83, *p* = 0.373; treatment × sex interaction: F_1, 22_ = 1.22, *p* = 0.282), nor when rats of both sexes were combined (*t* = 0.48, *df* = 24, *p* = 0.318 vs. saline-treated rats; Figure 2B). Finally, when evaluating total distance travelled (cm), a significant effect of sex was observed (F_1, 22_ = 14.43, *p* = 0.001), with no effect of treatment (F_1, 22_ = 0.47, *p* = 0.502) or treatment × sex interaction (F_1, 22_ = 0.07, *p* = 0.791). This effect was driven by the overall higher distance travelled for females (+994 ± 262 cm, ###*p* = 0.001 vs. male rats; Figure 2C). As expected, cocaine did not alter the distance travelled when rats of both sexes were combined (*t* = 0.56, *df* = 24, *p* = 0.290 vs. saline-treated rats; Figure 2C). Figure 2D shows a representative heat map of the time spent in each compartment for all treatment groups.

### Increased cocaine reward as assessed by a single exposure place preference paradigm in adulthood by the combined exposure of ethanol and cocaine in adolescence

A single cocaine exposure was administered to all rats during adulthood and following persistent withdrawal to assess the impact of a prior adolescent drug experience on cocaine reward (Figure 3). When evaluating the % time rats spent in the cocaine-paired compartment, the results showed no significant effects of treatment (F_3, 37_ = 2.54, *p* = 0.072), sex (F_1, 37_ = 0.79, *p* = 0.380) or treatment × sex interaction (F_3, 37_ = 1.09, *p* = 0.366; Figure 3A). When combining rats of both sexes, a one-way ANOVA detected a significant effect of the adolescent treatment (F_3, 41_ = 2.96, p = 0.043). Particularly, a cocaine challenge during adulthood increased the time rats treated with a combined mixture of ethanol and cocaine in adolescence spent in the drug-paired compartment (+7.4 ± 3.1%, *p = 0.05 vs. adolescent vehicle-treated rats; Figure 3A). Exposure to either ethanol or cocaine alone during adolescence did not increase the impact of a challenge dose of cocaine in adulthood (Figure 3A). When evaluating the number of entries in the cocaine-paired chamber, a two-way ANOVA found significant effects of the adolescent treatment (F_3, 36_ = 6.05, *p* = 0.002) and sex (F_1, 36_ = 8.57, *p* = 0.006), but no treatment × sex interaction (F_3, 36_ = 2.70, *p* = 0.060). The effect of sex was driven by the overall higher number of entries in the paired compartment done by females (+3.8 ± 1.3 number of entries, ##*p* = 0.006 vs. male rats; Figure 3B). When combining rats of both sexes, a one-way ANOVA detected a significant effect of the adolescent treatment (F_3, 40_ = 4.43, p = 0.009). Particularly, a cocaine challenge during adulthood increased the number of entries in the compartment paired with cocaine, but exclusively in rats treated with a combined mixture of ethanol and cocaine in adolescence (+7.3 ± 2.0 entries, **p = 0.003 vs. adolescent vehicle-treated rats; Figure 3B). Finally, when evaluating total distance travelled (cm), a significant effect of sex was observed (F_1, 37_ = 15.94, *p* < 0.001), with no effect of adolescent treatment (F_3, 37_ = 2.43, *p* = 0.081) or treatment × sex interaction (F_3, 37_ = 0.46, p = 0.715). As in the previous experiment, the sex effect was driven by the overall higher distance travelled for females (+946 ± 237 cm, ###*p* < 0.001 vs. male rats; Figure 3C). Figure 3D shows a representative heat map of the time spent in each compartment for all treatment groups.

## Discussion

The present study examined the impact of combining different drugs of abuse (ethanol and cocaine) during a vulnerable window in adolescence on the long-term changes on cocaine reward emerging throughout withdrawal in adulthood. Cocaine reward was assessed in adult rats by a simple paradigm based on a single exposure place preference previously characterized in mice, which was validated for Sprague-Dawley male and female adult rats. The main results demonstrated that combining ethanol with cocaine during adolescence increased the rewarding response induced by cocaine in adulthood when compared to rats that received no drugs in adolescence. These results also suggested that each drug might be producing partial effects or could have stronger effects if higher doses or different dosing regimens were tested. In conjunction, the combination of drugs during adolescence increased the susceptibility to addictive-like responses caused by drug re-exposure in adulthood as compared to rats with no prior drug exposure in adolescence, thus presenting an augmented risk-factor for later consequences in adult rats of both sexes.

While looking for a simple yet reliable protocol to assess the initial rewarding effects of cocaine in adulthood, we adapted a prior study based on a single exposure place preference in mice (Runegaard et al., 2017) to adult Sprague-Dawley rats of both sexes. This paradigm provided a reliable and convenient approach to assess cocaine reward in a novel environment, while avoiding the need for repeated drug injections (Runegaard et al., 2017) and was also validated for amphetamine reward (Runegaard et al., 2019). Our results proved that a single cocaine injection (15 mg/kg) was capable of inducing place preference, as observed by an increase in the time a group of adult rats of both sexes spent in the drug-paired chamber (55% preference for paired-chamber) as compared to rats injected with a saline solution (preference below 50%). As in previous recent experiments from our group (e.g., Colom-Rocha et al., 2024; Jornet-Plaza et al., 2025), female rats were more active/explorative when placed in a novel environment than males. In conclusion, we proved that a single exposure to cocaine was enough to induce place preference in adult Sprague-Dawley rats, serving as an indirect measure of the initial rewarding effects of cocaine. As discussed in more detail by Runegaard et al. (2017), this paradigm resembled prior one-trial place preference protocols, but shortened, without habituation and initial preference testing, allowing the evaluation of the initial rewarding effects of cocaine within just a few days. In our hands it therefore provided a simple procedure in which to test the long-term impact of combining different drugs of abuse (ethanol and cocaine) during a vulnerable window in adolescence on the rewarding properties of cocaine in adulthood following persistent withdrawal.

When adolescent rats were exposed in adolescence to a single drug, either ethanol or cocaine, no change was observed in the expected rewarding effects of a single cocaine dose in adulthood (i.e., 56% and 54% preference, respectively; note that all rats were paired with cocaine, thus these values were similar to the ones observed in the first part of this study). Prior literature suggested that ethanol exposure during adolescence increased the abuse liability of cocaine in adulthood, since animals were sensitized, and lower doses of cocaine were needed to be perceived as rewarding (e.g., Hutchison and Riley, 2012). However, with our non-contingent paradigm of adolescent ethanol exposure, a single exposure of cocaine in adult rats did not increase its expected rewarding response. Contrarily, the lack of change in the rewarding effects of cocaine in adulthood by adolescent cocaine aligned with prior literature suggesting that cocaine exposure during adolescence might desensitize adult animals raising the rewarding threshold necessary to drive place preference (i.e., higher cocaine doses), suggesting the development of a higher vulnerability during adulthood (Caffino et al., 2021; reviewed by Steinfeld et al., 2023). These results reinforce the need for future studies evaluating cocaine reward at higher doses in adulthood, to better comprehend how ethanol or cocaine might alter this response, as well as the potential cross-effects among drugs. Overall, although these data seemed contradictory in terms of later liability (i.e., sensitized vs. desensitized responses), it reinforced the idea that an early initiation to drug exposure induced long-term consequences in rodents, as detailed in the previous literature (e.g., Stansfield and Kirstein, 2007; García-Cabrerizo et al., 2015; García-Fuster et al., 2017; García-Cabrerizo and García-Fuster, 2019; Carrara-Nascimento et al., 2020; Parsegian et al., 2022) and as reviewed throughout the years by several groups (e.g., Spear, 2016; Salmanzadeh et al., 2020; Steinfeld et al., 2023).

Interestingly, when ethanol and cocaine were combined during adolescence, the main results demonstrated a vulnerable outcome in adulthood, as observed by the increased place preference response induced by a single cocaine challenge (i.e., 62% preference). Therefore, combining ethanol and cocaine during adolescence increased the lack of response of each individual drug as compared to rats that received no drugs, suggesting an elevated risk-factor for later consequences in adulthood, both in male and female rats. These findings contribute to the existing literature in the field (reviewed by Crummy et al., 2020; Steinfeld et al., 2023) by increasing the number of studies ascertaining the effects of drugs in combination to better model human consumption, especially during adolescence, since early drug initiation can be predictive of abuse later in life. Our data is a clear example that combining ethanol with cocaine early on in life increased the individual susceptibility to addictive-like responses caused by drug re-exposure in adulthood as compared to rats with no prior drug exposure in adolescence. Future studies should evaluate whether higher doses and/or different dosing regimens could induce stronger effects for each individual drug. Another key avenue relies on ascertaining the molecular mechanisms driving this combined vulnerability. One potential mechanism to further explore is associated with the impairments of hippocampal function and structure, such as the different stages of hippocampal neurogenesis, which we have recently explored across time in the context of ethanol alone and/or combined with adolescent cocaine exposure (see Colom-Rocha and García-Fuster, 2025). Ongoing research is essential to fully comprehend the effects of polysubstance use and how it impacts the brain and behavior. In this line of thought, our group has also proven that the combination of ethanol and cocaine in adolescence did not induce a higher impact vs. just ethanol alone when the vulnerability factor evaluated in adulthood centered in a paradigm of voluntary ethanol consumption (see Colom-Rocha et al., 2024) suggesting specific cross-effects when combining these drugs in adolescence depending on the outcome evaluated later in adulthood.

The present results are limited by the conditions tested and might have benefited from a full dose effect curve analysis for the cocaine place-preference response. Additionally, the protocol used for single exposure place-preference was slightly modified from the original (Runegaard et al., 2017; Runegaard et al., 2019), which means that results obtained may differ from those derived using the original protocol. In addition, only a single dose of ethanol and/or cocaine was used during the adolescent treatment phase. It is certainly possible that other dosing regimens may have produced different results. In any case, these doses were selected from prior studies from our group that had proven changes in behavioral and neurochemical outcomes (García-Cabrerizo et al., 2015; García-Cabrerizo and García-Fuster, 2016; García-Fuster et al., 2017; García-Cabrerizo and García-Fuster, 2019; Parsegian et al., 2022; Colom-Rocha et al., 2024). Future studies should therefore center in further ascertaining the impact of other doses of ethanol and cocaine in adolescence, as well as exploring how adolescent exposure to ethanol and cocaine increases the rewarding effect of cocaine in adulthood. Moreover, the sample size was too small to ensure reliable results for each sex separately, thus suggesting the need for a larger sample to compare the data between male and female rats effectively in upcoming experiments.

## Conclusion

Overall, our results demonstrate that co-exposure to ethanol and cocaine during adolescence has lasting effects, enhancing the sensitivity to cocaine reward in adulthood for both sexes. These findings highlight the potential long-term risks associated with early polysubstance use and reinforce the importance of considering combined drug exposure in models of addiction vulnerability.

## Data Availability

The raw data supporting the conclusions of this article will be made available by the authors, without undue reservation.
